# Books and Authors

**Published:** 1907-09

**Authors:** 


### BOOKS AND AUTHORS.

**Diseases of the Lungs.** By **Robert H. Babcock,** A.M., M.D., author of Diseases of the Heart and Arterial System. Octavo, pp. 829. With twelve colored plates and 104 text illustrations. New York and London: D. Appleton and Co. 1907. (Price $6.00).

The author of this work gained for himself a worldwide fame through his treatise on diseases of the heart and arterial system issued in 1903. An examination of this work leads to the conclusion that it will take its place alongside of the other, and that the two together may easily be pronounced the most learned exposition of the diseases of the organs of the chest that has been written by an American physician.

It is evident that the work is written by a clinician of experience and will find its way promptly into the hands of other clinicians. Babcock is also familiar with the literature of his subject, and does not hesitate to point out valuable suggestions therefrom. Since Flint, in the forties, delineated the sounds of the breathing organs in health and disease, many other clinicians have studied and written upon the subject, but none within our knowledge has developed the finer points of physical exploration of the chest to the extent that Babcock has done.

It would be interesting to follow this author all through his book, chapter by chapter, directing attention here and there to special points that are emphasised or developed, created or accentuated, but we could hardly expect an audience in this vacation season to read or heed such an extended commentary. We must rather content ourselves, and thus content our readers, by directing attention to one or two strong,—we may say with great propriety, stronger,—features of this treatise.

Babcock begins his book with a section on diseases of the bronchial tubes; in other words he takes up his studies just at the point where diseases of the upper air tract leave off. In the olden time, that is to say before the days of the specialism of laryngology, diseases of all the breathing organs were dealt with by one and the same author. Nowadays, however, the area which the teacher of internal medicine handles begins with the bronchi, and we desire to call attention to the skill with which the topic is treated in this book. The author very properly animadverts upon the cigarette habit, believing that it is a factor in causing disease of the bronchial tract. Babcock's ideas of treatment of the various diseases of the bronchial tubes are modern in every way and appeal to the better judgment of every experienced clinician.

In the second section diseases of the lungs are considered, and we are pleased with the way in which the pneumonias are dealt with. It is doubtful if acute fibrous pneumonia has ever received a more scientific consideration than in the five chapters which Babcock devotes to this topic. The same might be said of pulmonary tuberculosis, for here, also, great praise is due for the thoroughness and scientific handling of the subject embracing, as he has, every modern thought in causation and treatment.

Diseases of the pleura occupy the third section, which consists of three chapters. Here again, the author shows acumen in discussing these affections from the viewpoint of the modern laboratory. The bacteriological origin of pleurisy has been clearly established, and three microorganisms are chiefly responsible for it—namely, the tubercle bacillus, the diplococcus pneumoniæ, and the streptococcus pyogenes. The first two are mainly found in primary pleurisies, especially the tubercle bacillus; while the streptococcus belongs principally to secondary pleurisies. In the light of recent investigations the notion of idiopathic pleuritis must be abandoned. We should like to pursue this subject further on the lines mapped out by Babcock, but are compelled to desist lest we exceed our allotted limits of time and space.

We must not fail to mention the illustrations, many of which are meritorious, especially the plates depicting malignant disease of the lungs. We have never seen a work on diseases of the chest with as many useful engravings as are found in this treatise.

---

**Paraffin in Surgery.** A Critical and Clinical Study by **Wm. H. Luckett,** M.D., Attending Surgeon, Harlem Hospital, Surgeon to the Mt. Sinai Hospital Dispensary of New York, and **Frank I. Horne,** M.D., Formerly Assistant Surgeon, Mt. Sinai Hospital Dispensary. 12 mo.; 38 illustrations; 118 pages. New York: Surgery Publishing Co., 92 William street. (Cloth, $2.00).

This large book dealing with a large subject, required two authors to set forth the topic in proper perspective. The illustrations are of the "before and after" sort but, it must be admitted, portray with graphic effect the paraffin treatment for facial deformities. Generally speaking, there are two sides to a question, and it is apparent that the use of paraffin in surgery presents no exception to this nearly infallible rule. These authors, recognising the dangers that may result from accidental effects or careless use of paraffin, have given in two chapters the unsatisfactory results and the accidents that may follow its employment.

The chemistry of paraffin, little understood perhaps by the average physician, is discussed in a short chapter which imparts sufficient information for all practical purposes. It takes an expert chemist, however, to fully appreciate all the technical details of this peculiar substance.

This book will do much good; it places the important facts relating to paraffin in surgery before the profession in accessible form, and is likely to prevent its harmful employment. We commend it to those interested in the subject.

---

**A Textbook upon the Pathogenic Bacteria.** For Students of Medicine and Physicians. By **Joseph McFarland,** M.D., Professor of Pathology and Bacteriology in the Medico-Chirurgical College, Philadelphia. New (5th) edition. Octavo volume of 647 pages, fully illustrated, a number in colors. Philadelphia and London: W. B. Saunders Company, 1906. (Cloth, $3.50 net).

A study of pathogenic bacteria is now a necessary part of the medical curriculum, and this work comes close to being a necessary part of the student's library, since there is no work of similar character that quite takes the place of it. McFarland has come to be acknowledged authority on the subject of which this volume treats, and the five editions of his book coming in such rapid succession bespeaks not only his own popularity as a teacher, but tells of the growing study of the microorganisms of disease.

Much new material has been added to this edition, while the chapters on infection and immunity have been entirely rewritten. It is a work of progress, each new issue recording the advances since the last and in this way keeping pace with the increased knowledge of the topic with which it deals.

---

**A Textbook of the Practice of Medicine.** For Students and Practitioners. By **Hobart Amory Hare,** M.D., B.Sc., Professor of Therapeutics and Materia Medica in the Jefferson Medical College of Philadelphia; Physician to the Jefferson Medical College Hospital; Author of A Textbook of Practical Therapeutics; A Textbook of Practical Diagnosis, etc. Octavo, 1120 pages, with 131 engravings and 11 full-page plates. Second edition, revised and enlarged. Philadelphia and New York: Lea Brothers & Co. 1907. (Cloth, $5.00; leather, $6.00; half morocco, $6.50, net prices).

As might have been expected, the first edition of this work was soon exhausted, and the author was called upon for the second edition with promptitude. The response has been equally prompt and we find ourselves called upon to make mention again in these columns of one of the most useful, because one of the most practical, of textbooks on medicine yet presented to the profession. Hare is among our best teachers of internal medicine, he is a popular writer, and his books are always in active demand.

Hare's practice of medicine presents itself in this new or second edition in the same excellent form that characterised the first; it is a book for the undergraduate and practitioner; it is especially designed to aid at the bedside, and is therefore strong in delineating modern methods of treatment; and, finally, it is one of the most compact, hence valuable ready-reference works on practice of which we have knowledge. It has been revised to include all that is valuable and to exclude all that is obsolete or valueless. It is authority on medical diseases.

---

**Principles and Application of Local Treatment in Diseases of the Skin.** By **L. Duncan Bulkley,** A.M., M.D., Physician to the New York Skin and Cancer Hospital. Small 8vo., 142 pages. New York: Rebman Co. (Price, $1.00).

This book is made up from four lectures given to practising physicians at the New York Skin and Cancer Hospital in the spring of 1906, which are published in this form because of requests by many who wish to see them in print. Dr. Bulkley is an experienced teacher, as well as writer and clinician; his writings are popular because they are practical; and, withal, he is a scientific dermatologist who gets results. In this small treatise the author merely attempts to expound the underlying principles and application of local treatment in diseases of the skin, but he has accomplished his purpose most happily. He gives to the general practitioner just the information he needs to enable him to cope with the simpler skin maladies, or to recognise the important time to recommend a patient to consult a dermatologist. At all events it is just such a book as every physician should possess and he should be guided by its teachings.

---

**The Nursling.** The Feeding and Hygiene of Premature and Full-term Infants. By **Pierre Budin,** Professor of Obstetrics, University of Paris; Director of the Clinique Tarnier. Authorised Translation by **William J. Maloney,** M.B., Ch. B., Fellow of the Obstetrical Society of Edinburgh, with an introduction by **Sir Alexander R. Simpson,** M.D., Emeritus Professor of Midwifery and Diseases of Women and Children, University of Edinburgh. Large 8 vo, pp. 223. With 111 diagrams in color and other illustrations. London: The Caxton Publishing Company. New York: Imperial Publishing Company. 1907. (Price, $6.00.)

The title of this book is a new one for a treatise on infant hygiene; nevertheless, it is expressive of the scope the author gives himself, since the book deals with infants from birth until they reach the age of two years. Pierre Budin is very well known on this side of the Atlantic as a teacher of great power. Alexander R. Simpson says that Professor Budin is a past master in the eloquent use of what he, Sir Alexander, is pleased to term the most lucid of modern languages. At all events these lectures are graphic descriptions of modern methods in dealing with infantile diseases that cause great havoc in summer time, and will be read with interest by every physician on this side of the water, whose province it is to deal with the summer diarrheas of infants. The prospectus of the publisher gives in concise form the essential purposes of the book stating, among other things, that one of its special features is the saving of children born before term, and that teething, weaning, and all problems relating to infant feeding are exhaustively dealt with.

The volume is beautifully printed on deckled paper, is illustrated by diagrams in color and otherwise, and is among the special features of the year in book making which serves to enrich the literature of the period.

---

**Medical Diagnosis.** A Manual of Clinical Methods for Practitioners and Students. By **J. J. Graham Brown,** M.D., F.R.C.P.E., F, R.S.E., Assistant Physician, Royal Infirmary of Edinburgh, and **W. T. Ritchie,** M.D., F.R.C.P.E., F.R.S.E., Clinical Assistant Pathologist, Royal Infirmary of Edinburgh. Fifth edition. 12 mo., pp. 524. With 200 illustrations and 8 full page plates. New York: Imperial Publishing Company. 1907. (Price, $3.00).

A book like this that has been issued in four editions needs but little comment on the fifth, except to speak in general commendation of such changes as have been made in the revision. It is evident on inspection that almost every section has been revised, many additions having been made, and that the book may be regarded as a thoroughly modern manual of diagnosis. The urinary system in particular receives ample treatment, full instructions for the estimation of urea and other products of nitrogenous metabolism being given, and tests for albumin, glucose and other outputs relating to destructive changes in organs and tissues. The entire book constitutes a clinical manual of unquestioned merit for the practitioner, and is a valuable guide for the studies of the undergraduate The illustrations are unusual in number for such a work, many being of exceptional merit.

---

**Aids to the Diagnosis and Treatment of Diseases of Children.** By **John McCaw,** M.D., Physician to the Belfast Hospital for Sick Children. Third edition. 383 pages. New York: William Wood & Co. (Price, $1.25).

The book under consideration is one of the best of the students' reminders that has been issued. McCaw is an experienced teacher, hence knows what students of medicine need most as an aid to the memory. This third edition has enabled the author to add new, as well as improve the old material. To the student of pediatrics this book will prove vastly useful, and even to some of the elder members of the profession it may serve as an acceptable reminder of partially forgotten facts in relation to the diseases of children.

**Students Aid Series. Aids to Dental Surgery.** By **Arthur S. Underwood,** M.R.C.S., L.D.S., Eng. And Douglas Gabell, M.R.C.S„ L.R.C.P., Lond., L.D.S., Eng. Second edition. 126 pp. New York: William Wood & Co. (Price, $1.00).

That the profession of dentistry should require an "aid" is indeed, evidence of progress. Only a few years ago dentistry, in the main, was acquired by an apprenticeship in a dentists office, and not a very long service at that. Now, dentistry is taught in well equipped colleges which often, as in Buffalo, are attached to universities, hence need textbooks, manuals, aids, and other literature. This book has been prepared by men familiar with the needs of dental students and this edition has been enriched with new chapters dealing with bacteriology and hygiene of the mouth. If illustrations had been inserted the volume would have been a little more complete, but, even now it is the best, if not the only book of its kind and will not disappoint the dental student who may purchase it.

---

**Students Aid Series. Aids to Medical Diagnosis.** By **Arthur Whiting,** M.D., M.R.C.P., Physician to the Tottenham Hospital; Lecturer in and Dean of the Northeast London Post-Graduate College. 152 pages. New York: William Wood & Co. (Price, $1.00).

Such books as this have become a necessity, at least practically so, in these days of rapidity of action and thought. To those who have acquired some knowledge of medicine this "aid" will prove serviceable in many ways. It is written by a practical teacher, a clinician of experience, and the basis adopted is clinical rather than pathological. The symptom complex is unraveled as far as possible in each group in logical sequence, and the round up leads to differentiation of each constituent, whenever it is possible to do so. The study of diagnosis will be facilitated by following the lines marked out by Whiting in this manual.

---

**A Manual of Obstetrics.** By **A. F. A. King,** M.D., Professor of Obstetrics and Diseases of Women in the Medical Department of the George Washington University, Washington, D. C., and in the Medical Department of the University of Vermont, etc. Tenth edition. 12mo., 688 pages, with 30 illustrations and three colored plates. Lea Brothers & Co., Philadelphia and New York, 1907. (Cloth, $2.75, net.)

This remarkable book, now in its tenth edition, will be welcomed by hosts of old friends, and should be greeted by many new ones. Dr. King is one of the most practical as well as one of the most accomplished teachers of obstetrics of the present day. Knowing what students need, he has put this information into a manual that has withstood the test of time, receiving alike the approbation of undergraduates, teachers, and practitioners, all of whom find it a most satisfactory book of reference, study, or preparation. In this edition additions have been made, errors corrected, and obsolete methods eliminated, thus bringing it forward to the immediate present and making it one of the best obstetric manuals in existence.

